# Frailty is associated with worse cognitive functioning in older adults

**DOI:** 10.3389/fpsyt.2023.1108902

**Published:** 2023-02-01

**Authors:** Chunmei Li, Song Ge, Yueheng Yin, Chong Tian, Yongxia Mei, Peijin Han

**Affiliations:** ^1^Infection Clinic, Shandong Provincial Hospital Affiliated to Shandong First Medical University, Jinan, China; ^2^Department of Natural Sciences, University of Houston-Downtown, Houston, TX, United States; ^3^School of Nursing, Nanjing Medical University, Nanjing, China; ^4^School of Nursing, Tongji Medical College, Huazhong University of Science and Technology, Wuhan, China; ^5^School of Nursing and Health, Zhengzhou University, Zhengzhou, Henan, China; ^6^Department of Computational Medicine and Bioinformatics, Medical School, University of Michigan, Ann Arbor, MI, United States

**Keywords:** cognitive functioning, frailty, older adults, NHANES, the fried phenotype

## Abstract

**Introduction:**

Frailty and impaired cognitive functioning often co-occur in older adults and are associated with adverse health outcomes. However, their relationship is unclear. This study sought to examine the association of frailty status with cognitive functioning in older adults.

**Method:**

The study population consisted of 2,296 older adults aged ≥60 from the National Health and Nutrition Examination Survey 2011–2014. Frailty status was measured based on the Fried Phenotype and the participants were categorized into three groups- robust, pre-frailty, and frailty. Cognitive functioning was measured using the Consortium to Establish a Registry for Alzheimer’s Disease Word Learning subtest (CERAD-WL) immediate and delayed recall tests, the Animal Fluency test (AFT), and the Digit Symbol Substitution Test (DSST). Test-specific and global cognition z-scores were calculated. Multinomial linear regression models were constructed to examine the association between frailty status (reference: robust) and test-specific and global cognition z-scores. Multiple linear regression models were used to examine the relationship between the number of frailty dimensions and test-specific and global cognition z-scores. All models controlled for age, race/ethnicity, education, total cholesterol level, and systolic blood pressure.

**Results:**

About half of the participants (median age 68 years) were female (49.9%) and non-Hispanic White (48.7%). A quarter (23.3%) of the participants completed some college and above. Multinominal linear regression showed that compared with participants who were robust, those with frailty had worse DSST [β = –0.234, 95% confidence interval (CI): –0.391, –0.078, *P* = 0.003] and global cognition z scores (β = –0.129, 95% CI –0.233, –0.025, *P* = 0.02). Multiple linear regression model showed that the number of frailty dimensions was significantly associated with decreased the DSST (β = –0.065, 95% CI –0.103, –0.026, *P* = 0.001) and global cognition z-scores (β= –0.034, 95% CI –0.06, –0.009, *P* = 0.009).

**Conclusion:**

Frailty is associated with worse processing speed, sustained attention, working memory, and global cognition in older adults. Prevention and treatment of frailty in older adults may help protect their cognitive functioning. Further, clinicians should consider assessing cognitive functioning, especially processing speed, sustained attention, and working memory, among frail older patients, which may allow early identification and interventions of cognitive impairment.

## Introduction

Pre-frailty and frailty are prevalent aging-related symptoms (1). Frailty is characterized by a compromised ability to respond to external stimuli. It is associated with a long-term cumulative decline in reserves and functions in multiple systems in the human body (2, 3). Pre-frailty is a latent condition that increases a person’s risk of frailty and typically occurs before clinically identifiable frailty (4). Existing studies have found that the prevalence of frailty among community-dwelling older individuals 65 and older ranges from 4.0 to 59.1% (1, 5). Compared with robust older adults, those with frailty are more likely to fall, be disabled, immobile, require hospitalizations, and have a lower quality of life (6). Thus, identifying and intervening in frailty in older adults is important to prevent, delay, reverse, or reduce frailty and its associated adverse outcomes (7–9).

Another area that concerns older adults is cognitive impairment. Cognitive impairment often co-occurs with frailty, causing increased disability, reduced quality of life, and higher morbi-mortality in this population (10). Frailty and cognitive decline are complicatedly related, although the biological origins of frailty have not yet been fully understood (11). A systematic review including fourteen studies revealed that few studies examined the association between frailty and specific domains of cognitive functioning (12). The assessment of cognitive functioning in previous studies mainly focused on the general measurement using either the Mini-Mental State Examination (MMSE), Montreal Cognitive Assessment (MoCA), or the Mini-Cog instrument. However, the manifestations of cognitive impairment are complicated and often fall into different cognitive domains, for example, literal deficit or decline in learning ability. Thus, it is difficult to show the specific cognitive impairment using the general cognitive scales (13). Therefore, the purpose of this study is to examine the association between frailty status and various cognitive functioning domains in older adults using the National Health and Nutrition Examination Study (14) 2011–2014. The findings of this study will provide implications for clinical practice and policymaking aiming at preventing frailty and cognitive impairment in older adults.

## Materials and methods

### The parent study design

The NHANES is a cross-sectional study of civilian, non-institutionalized U.S. populations, including both adults and children. The NHANES is conducted by the National Center for Health Statistics of the Centers for Disease Control and Prevention (CDC) (NHANES) biannually. About 5,000 participants from diverse sociodemographic regions across the US are recruited using a complex, multistage probability strategy in every 2 years cycle (15). Data, including sociodemographic, health, and nutritional information, are collected using in-person home interviews and health exams at mobile exam centers. Cognitive functioning was assessed only in participants aged ≥60. We merged the NHANES (14) 2011–2012 (*n* = 9,338) and 2013–2014 (*n* = 9,813) to increase power. We excluded people who had missing frailty status or cognitive functioning (*n* = 16,855). Finally, the study population consisted of 2,296 older adults aged 60 and above.

### Ethical considerations

The NHANES gained ethical approval from the National Center for Health Statistics Research Ethics Review Board. The University of Houston-Downtown Committee for the Protection of Human Subjects exempted this present study.

### Measures

#### Independent variable: Frailty status (robust, pre-frailty, or frailty)

Frailty status was defined based on the Fried Phenotype (4) and following a previous NHANES study (16). If three or more of the following conditions were met, the participant was determined to be frail, including unintended weight loss, sluggish walking, weakness, fatigue, and a lack of physical activity. If a participant met one or two of the above conditions, he was determined to be pre-frail. If none of the criteria was present, the participant was robust. Our definition followed the five dimensions of the Fried Phenotype; however, we had to modify the standards since we relied on the available NHANES data. The modification was consistent with a previously published study (16).

(1) Unintentional weight loss was measured by three questions: (a) “How much do you weigh without clothes or shoes?” (b) “How much did you weigh a year ago?” and (c) “was the change between your current weight and weight a year ago intentional?” Low body weight for height was defined as having a body mass index (BMI) **≤** 22.5 kg/m^2^ or at least 5% unintentional weight loss (responding that their weight loss was not intentional) in the past year. Other published studies have used the same criteria to operationally measure this concept (17, 18).
(2) Sluggish walking. For this question, “by yourself and without using any special equipment, how much difficulty do you have walking from one room to another on the same level?,” if participants responded, “with some difficulty,” “with significant difficulty,” or “unable to do,” they would be categorized as having sluggish walking.
(3) Weakness. For this question, “by yourself and without using any special equipment, how much difficulty do you have lifting or carrying something as heavy as 10 pounds?,” if participants responded, “with some difficulty,” “great difficulty,” or “unable to do,” they would be categorized as having weakness.
(4) Fatigue. For this question, “by yourself and without using any special equipment, how much difficulty do you have walking for a quarter of a mile?,” if participants responded, “with some difficulty,” “great difficulty,” or “unable to do,” they would be categorized as having fatigue.
(5) Lack of physical activity. After participants reported an average amount of vigorous and moderately intense activity in minutes for (a) work, (b) going to and from locations, and (c) recreation, we calculated metabolic equivalent (MET) minutes. MET is a measurement of how much energy is expended by a person performing a given physical activity in relation to their mass when compared to a static reference. One MET minute equals the amount of oxygen consumed while sitting at rest (19). Participants would be categorized as lack of physical activity if their MET minutes per week were below 600.

#### Dependent variable: Cognitive functioning

We used the following cognitive tests to assess cognitive functioning, including the Consortium to Establish a Registry for Alzheimer’s Disease Word Learning subtest (CERAD-WL) immediate and delayed recall test, the Animal Fluency test (AFT), and the Digit Symbol Substitution test (DSST).

(1) The CERAD-WL assesses a person’s ability to use immediate and delayed memory to obtain verbal knowledge (20). The CERAD-WL included three rounds of learning trials and a delayed recall test. In each learning trial, a person was asked to read out ten, one at a time, random words in large boldface on a computer monitor. A person was required to memorize and recall as many words as possible right after the words were presented. The sequence of the words was different over the three trials, so a maximum score of 10 was possible on each trial. The total score of three trials reflected a participant’s immediate memory score and ranged from 0 to 30. In addition, a delayed recall test was conducted after the AFT and the DSST. In the delayed recall test, the person needed to speak out as many words as he/she could from the afront-mentioned 10-word list. The correct number of recalled words was his/her delayed memory score which ranged from 0 to 10. The CERAD-WL has been widely used in epidemiological studies (21–23).
(2) The AFT measures a person’s fluency in language, a part of the executive functioning (24). A person was asked to name as many animals as he/she could in 60 s and was awarded one score for each correct animal identified. The AFT has been shown to effectively differentiate mild cognitive impairment and probable Alzheimer’s disease in older adults (25). It has also been widely used in epidemiological studies (26).
(3) The DSST is a performance module from the Wechsler Adult Intelligence Scale (WAIS-III) and assesses a person’s processing speed, sustained attention, and working memory (27). The test was administered using a paper form with a top-mounted key containing nine numbers and their paired symbols. The 133 boxes were next to the numbers containing corresponding symbols, and the person had 2 min to match the symptoms to the boxes. The number of correct matches was the person’s score (28), which ranged from 0 to 133 (29). The DSST has been widely used in epidemiological studies (30, 31).

#### Covariates

To control for confounding between frailty status and cognitive functioning, we reviewed the literature (8, 9, 32) and the following covariates were included in the analysis- age (years), sex (male or female), race/ethnicity (Mexican Americans, other Hispanics, non-Hispanic White, or non-Hispanic Black), education (below high school, high school graduate, or some college or above), total cholesterol (mg/dL), and systolic blood pressure (mmHg).

#### Statistical analysis

For descriptive statistics, means [standard deviation (SD)] were used to describe continuous data with normal distribution and medians (interquartile range) for continuous data not following a normal distribution. Frequency (percentages) was used to describe categorical data. Using means and standard deviations of the cognitive test scores, test-specific z-scores were calculated for the CERAD-WL immediate memory, the CERAD-WL delayed memory, the AFT, and the DSST, respectively. Global cognition z-score was then calculated by averaging all test-specific z-scores. One-way ANOVA and χ^2^ tests were performed to examine group differences for continuous and discrete variables, respectively, in three frailty status groups.

Multinomial linear regression models were used to examine the independent relationship between frailty status (reference: robust) and test-specific and global cognition z-scores, controlling the covariates mentioned above. A 95% confidence interval (CI) excluding zero or a *P*-value < 0.05 was considered statistical significance. All analyses were performed using R version 4.0.3.

## Results

The characteristics of the study population were presented in Table 1. The 2,296 participants had a median age of 68.0 years with an interquartile range from 63 to 74. About half of them were female (49.9%) and non-Hispanic White (48.7%). A quarter of the participants (23.3%) completed some college or above; 31.4% had 0–1 alcoholic drink per day. The participants had a median of three depression symptoms measured by the Patient Health Questionnaire-9 (interquartile range 2–6). They had a median of 191 mg/dL total cholesterol (interquartile range 163.0–220.0) and 132.0 mmHg systolic blood pressure (interquartile range 120.0–144.0). In terms of their frailty status, most of them had pre-frailty (69.3%), followed by robust (25.6%), and frailty (5.1%).

**TABLE 1:** Characteristics of the participants by frailty status.

Variables	Robust (*n* = 587)	Pre-frailty (*n* = 1592)	Frailty (*n* = 117)	Total (*n* = 2296)	*P*-value^d^
Age, years^a^	68 (63, 74)	68 (63, 74)	69 (63, 77)	68 (63, 74)	0.34
Sex, *n* (%)^b^	–	–	–	–	**<0.001**
Male	299 (50.9%)	816 (51.3%)	35 (29.9%)	1150 (50.1%)	–
Female	288 (49.1%)	776 (48.7%)	82 (70.1%)	1146 (49.9%)	–
Race/ethnicity, *n* (%)	–	–	–	–	0.48
Mexican Americans	53 (9%)	129 (8.1%)	11 (9.4%)	193 (8.4%)	–
Other Hispanics	62 (10.6%)	160 (10.1%)	7 (6%)	229 (10.0%)	–
Non-Hispanic Whites	278 (47.4%)	774 (48.6%)	66 (56.4%)	1118 (48.7%)	–
Non-Hispanic Blacks	141 (24%)	354 (22.2%)	24 (20.5%)	519 (22.6%)	–
Other	53 (0.9%)	175 (11%)	9 (7.7%)	237 (10.3%)	–
Education, *n* (%)	–	–	–	–	**0.02**
Below high school	140 (23.9%)	354 (22.2%)	40 (34.2%)	534 (23.3%)	–
High school graduate	152 (25.9%)	355 (22.3%)	29 (24.8%)	1224 (53.3%)	–
Some college or above	295 (50.3%)	881 (55.3%)	48 (41%)	536 (23.3%)	–
Alcoholic drinks/day, *n* (%)	–	–	–	–	**0.04**
0–1 drink	186 (31.7%)^c^	510 (32%)	25 (21.4%)	721 (31.4%)	–
2 drinks	92 (15.7%)	251 (15.8%)	14 (12%)	357 (15.5%)	–
3 or more drinks	68 (11.6%)	177 (11.1%)	10 (8.5%)	255 (11.1%)	–
Depressive symptoms	3 (2, 5)	3 (1, 5)	5 (2, 8)	3 (2, 6)	**<0.001**
Total cholesterol, mg/dL	192 (164, 222)	190 (163, 219)	192 (159, 222)	191 (163, 220)	0.31
Systolic blood pressure, mmHg	130 (120, 144)	132 (120, 144)	132 (120, 146)	132 (120, 144)	**0.05**
CERAD-WL immediate recall	20 (16, 23)	20 (17, 22)	19 (15, 22)	20 (16, 22)	0.57
CERAD-WL immediate recall z-score	0.198 (–0.557, 0.858)	0.198 (–0.557, 0735)	0.089 (–0.683, 0.735)	0.198 (–0.557, 0.735)	0.44
CERAD-WL delayed recall	0 (0, 0)	0 (0, 0)	0 (0, 0)	0 (0, 0)	0.97
CERAD-WL delayed recall z-score	–0.365 (–0.445, –0.365)	–0.365 (–0.445, –0.365)	–0.365 (–0.445, –0.365)	–0.365 (–0.445, –0.365)	0.97
AFT	16 (13, 20)	17 (13, 20)	16 (12, 20)	16 (13, 20)	**0.02**
AFT z-score	–0.076 (–0.624, 0.607)	0.058 (–0.624, 0.653)	–0.125 (–0.856, 0.607)	–0.077 (–0.624, 0.653)	**0.02**
DSST	49 (36, 61)	49 (37, 60)	44 (30, 54)	48 (36, 60)	**<0.001**
DSST z-score	0.168 (–0.575, 0.869)	0.168 (–0.518, 0.851)	–0.125 (–0.86, 0.46)	0.167 (–0.534, 0.851)	**<0.001**
Global cognitive z-score	–0.107 (–0.456, 0.411)	0.080 (–0.315, 0.490)	0.055 (–0.348, 0.461)	0.065 (–0.331, 0.479)	**<0.001**

^a^Data were presented as median (interquartile range) for continuous variables.

^b^Data were presented as *n* (%) for categorical variables.

^c^Column percentages did not sum to 100% because of missingness.

^d^One-way ANOVA test.

Bolded values mean statistical significance (*P* < 0.05). CERAD-WL, Consortium to Establish a Registry for Alzheimer’s Disease Word Learning subtest; AFT, animal fluency test; DSST, digit symbol substitution test.

Multinominal linear regression (Table 2) showed that compared with participants who are robust, those with frailty had worse DSST (β = –0.234, 95% CI –0.391, –0.078, *P* = 0.003) and global cognition z-scores (β = –0.129, 95% CI –0.233, –0.025, *P* = 0.02). No significant associations were found between frailty status and the CERAD-WL immediate, the CERAD-WL delayed memory, or the AFT z-scores. Compared to participants who are robust, pre-frailty was not significantly associated with test-specific or global cognitive z-scores.

**TABLE 2:** The associations between frailty and test-specific and global cognitive z-score.

		Dependent variables									
Independent variable		CERAD-WL immediate recall	*P*	CERAD-WL delayed recall	*P*	AFT	*P*	DSST	*P*	Global cognition	*P*
Frailty	Robust	Ref	–	Ref	–	Ref	–	Ref	–	Ref	–
	Pre-frail	0.009 (–0.079, 0.097)	0.84	–0.015 (–0.107, 0.078)	0.76	0.038 (–0.051, 0.127)	0.40	–0.01 (–0.083, 0.063)	0.79	0.006 (–0.043, 0.054)	0.82
	Frail	–0.137 (–0.325, 0.051)	0.15	0.002 (–0.197, 0.201)	0.98	–0.147 (–0.338, 0.044)	0.13	–0.234 (–0.391, –0.078)	**0.003**	–0.129 (–0.233, –0.025)	**0.02**
Age, years		–0.037 (–0.044, –0.031)	**<0.001**	0.01 (0.003, 0.016)	**0.004**	–0.035 (–0.041, –0.028)	**<0.001**	–0.046 (–0.051, –0.041)	**<0.001**	–0.027 (–0.031, –0.024)	**<0.001**
Sex, female		–0.402 (0.322, 0.481)	**<0.001**	–0.102 (0.186, –0.018)	**0.02**	–0.069 (–0.149, 0.012)	0.10	0.293 (0.227, 0.359)	**<0.001**	0.131 (0.087, 0.175)	**<0.001**
Race/ethnicity	Mexican Americans	0.014 (–0.170, 0.199)	0.88	0.129 (–0.066, 0.324)	0.19	0.519 (0.333, 0.705)	**<0.001**	–0.331 (–0.484, –0.177)	**<0.001**	0.083 (–0.018, 0.184)	0.11
	Other Hispanics	–0.15 (–0.321, 0.020)	0.08	0.292 (0.112, 0.472)	**0.002**	0.331 (0.159, 0.503)	**<0.001**	–0.571 (–0.712, –0.429)	**<0.001**	–0.025 (–0.118, 0.069)	0.61
	Non-Hispanic Whites	0.175 (0.042, 0.308)	0.01	0.054 (–0.086, 0.194)	0.45	0.729 (0.595, 0.863)	**<0.001**	0.201 (0.09, 0.311)	**<0.001**	0.29 (0.216, 0.363)	**<0.001**
	Non-Hispanic Blacks	0.102 (–0.045, 0.248)	0.17	0.306 (0.152, 0.461)	**<0.001**	0.163 (0.015, 0.311)	**0.03**	–0.463 (–0.585, –0.342)	**<0.001**	0.027 (–0.054, 0.107)	0.51
	Other	Ref	–	Ref	–	Ref	–	Ref	–	Ref	–
Education	Below high school	–	**<0.001**	0.148 (0.041, 0.255)	0.007	–0.618 (–0.720, –0.515)	**<0.001**	–0.996 (–1.081, –0.912)	**<0.001**	–0.504 (–0.560, –0.448)	**<0.001**
	High school graduate	–0.250 (–0.345, –0.115)	**<0.001**	–0.045 (–0.146, 0.055)	0.38	–0.4 (–0.496, –0.303)	**<0.001**	–0.362 (–0.441, –0.283)	**<0.001**	–0.264 (–0.317, –212)	**<0.001**
	Some college or above	Ref	–	Ref	–	Ref	–	Ref	–	Ref	–
Total cholesterol, mg/dL		0.001 (0.000, 0.002)	0.21	0.001 (0.000, 0.002)	0.12	0.002 (0.01, 0.003)	**<0.001**	0.001 (0.000, 0.002)	**0.02**	0.001 (0.001, 0.002)	**<0.001**
Systolic blood pressure, mmHg		–0.003 (–0.005, –0.001)	**0.005**	0.000 (–0.002, 0.002)	0.99	–0.002 (–0.004, 0)	0.06	–0.003 (–0.005, –0.001)	**<0.001**	–0.002 (–0.003, –0.001)	**<0.001**

Bolded values mean statistical significance (*P* < 0.05). CERAD-WL, Consortium to Establish a Registry for Alzheimer’s Disease Word Learning subtest; AFT, animal fluency test; DSST, digit symbol substitution test.

We also examined the associations between the number of frailty dimensions (continuous variable) and cognitive functioning. We found that the number of frailty dimensions was significantly associated with decreased the DSST (β = –0.065, 95% CI –0.103, –0.026, *P* = 0.001) and global cognition z-scores (β=−0.034, 95% CI –0.06, –0.009, *P* = 0.009). All these relationships were independent of age, sex, race/ethnicity, education, total cholesterol level, and systolic blood pressure.

## Discussion

In this group of 2,296 older adults, we found that $\frac{3}{4}$ of them had either pre-frailty or frailty. Compared to robust, frailty was independently associated with worse DSST and global cognition z-scores. Moreover, the number of frailty dimensions was independently associated with worse DSST and global cognition z-scores. Our results indicated that prevention and intervention of frailty in older adults may help protect their cognitive functioning. Further, our findings also suggested that tailored instruments should be investigated and implemented to assess cognitive functioning among frail older adults, which may achieve early identification and interventions for cognitive impairment. Overall, our study provided implications for healthcare providers and policymakers aiming at preventing frailty and cognitive impairment among the growing older adults in the world.

The possible mechanism that explains the relationship between frailty and cognitive functioning is unclear but may be multifactorial. Researchers have found that frailty and cognitive impairment share common pathological pathways. Specifically, Alzheimer’s disease, reduced testosterone, diet Sarcopenia, chronic inflammation, cardiovascular risk, and mental health affect both physical and cognitive functioning (33). Moreover, chronic inflammation, impaired HPA stress response, imbalanced energy metabolism, endocrine dysregulation, mitochondrial dysfunction, oxidative stress, genomic markers, and metabolomic markers may play a role in physical frailty and cognitive decline (34). Given the multifactorial pathology process, strategic approaches are needed to prevent frailty and cognitive decline in the older population (34).

We admitted that we did not adjust for depression symptoms in our study. Studies have shown that frailty and depression often co-exist and reciprocally interact with each other (35–37). Prospective studies have demonstrated that depression might be a negative consequence of frailty (38, 39). Since frailty usually indicates ongoing physical decline, frail older adults may experience elevated emotional stress (36). On the other hand, depression may increase frailty by impairing physical health (37–39). In addition, many studies showed that depression is an important independent risk factor for cognitive decline and dementia (40–42). Although solid evidence is not yet identified, we suspect that depression may mediate the association between frailty and cognitive functioning. Therefore, we chose not to adjust depressive symptoms in our study. In future studies, the role of depression in the relationship between frailty and cognitive functioning should be investigated.

Although this study and another study (43) utilized the same population, their research question is quite different. In need, many studies on a wide variety of topics are published using the NHANES data every year. The strengths of this study are as follows. Firstly, cognitive functioning was measured using three cognitive tests and global cognition z-scores were computed. These tests cover various domains of cognitive functioning, including immediate recall, delayed recall, verbal fluency, processing speed, sustained attention, and working memory. Compared to commonly used instruments in clinical settings, such as the MMSE, the MoCA and the Mini-Cog, the DSST is associated with physical functioning with a stronger magnitude (44). According the results of our study, the DSST may be a more sensitive screening instrument when evaluating cognitive functioning among older adults with frailty. Secondly, we investigated this association in a diverse population consisting of 18.4% Hispanics and 22.6% Non-Hispanic Blacks. In addition, we adjusted sociodemographic and physical health factors to reduce the risk of residual confounding.

At the same time, several limitations should be admitted. To start, because of the cross-sectional nature of this study, we are unable to determine the temporal connection between frailty status and cognitive functioning. Furthermore, the median age of our study population was 68 years old and the prevalence of frailty was 5.1%, indicating that our study population is relatively younger and more robust compared with the general older population. Thus, the generalizability of this study is limited. In addition, we did not assess whether depression symptoms mediated the relationship between frailty status and cognitive functioning. The mechanisms explaining the relationship between these variables should be explored in future studies.

The following are the clinical implications of this study: frailty was found to be associated with worse cognitive functioning in older adults. In clinical settings, to prevent older adults from developing frailty and cognitive impairment, clinicians should assess patients’ frailty status and intervene in those with pre-frailty and frailty. Further, clinicians should consider using tailored screening tools to assess cognitive functioning among frail older patients, which may allow early identification and interventions of cognitive impairment.

## Data availability statement

The datasets presented in this study can be found in online repositories. The names of the repository/repositories and accession number(s) can be found below: The data that support the findings of this study are openly available on the NHANES website and can be accessed at https://www.cdc.gov/nchs/nhanes/index.htm.

## Ethics statement

The studies involving human participants were reviewed and approved by University of Houston-Downtown Committee for the Protection of Human Subjects. The patients/participants provided their written informed consent to participate in this study.

## Author contributions

CL, PH, SG, YM, YY, and CT drafted the initial manuscript, designed the study, and searched for literature. PH conducted the statistical analysis. All authors contributed to the critically revised the manuscript and approved the submitted version.
